# Mental stress recognition on the fly using neuroplasticity spiking neural networks

**DOI:** 10.1038/s41598-023-34517-w

**Published:** 2023-09-11

**Authors:** Mahima Milinda Alwis Weerasinghe, Grace Wang, Jacqueline Whalley, Mark Crook-Rumsey

**Affiliations:** 1 School of Engineering, Computer and Mathematical Sciences, Auckland University of Technology, Auckland, New Zealand; 2 Brain-Inspired AI and Neuroinformatics Lab, Department of Data Science, Sri Lanka Technological Campus, Padukka, Sri Lanka; 3 School of Psychology and Wellbeing, University of Southern Queensland, Toowoomba, Australia; 4 Centre for Health Research, University of Southern Queensland, Toowoomba, Australia; 5 Department of Computer Science and Software Engineering, Auckland University of Technology, Auckland, New Zealand; 6 Department of Basic and Clinical Neuroscience, King’s College London, London, UK; 7 UK Dementia Research Institute, Centre for Care Research and Technology, Imperial College London, London, UK

**Keywords:** Computer science, Brain imaging, Human behaviour

## Abstract

Mental stress is found to be strongly connected with human cognition and wellbeing. As the complexities of human life increase, the effects of mental stress have impacted human health and cognitive performance across the globe. This highlights the need for effective non-invasive stress detection methods. In this work, we introduce a novel, artificial spiking neural network model called Online Neuroplasticity Spiking Neural Network (O-NSNN) that utilizes a repertoire of learning concepts inspired by the brain to classify mental stress using Electroencephalogram (EEG) data. These models are personalized and tested on EEG data recorded during sessions in which participants listen to different types of audio comments designed to induce acute stress. Our O-NSNN models learn on the fly producing an average accuracy of 90.76% (σ = 2.09) when classifying EEG signals of brain states associated with these audio comments. The brain-inspired nature of the individual models makes them robust and efficient and has the potential to be integrated into wearable technology. Furthermore, this article presents an exploratory analysis of trained O-NSNNs to discover links between *perceived* and *acute* mental stress. The O-NSNN algorithm proved to be better for personalized stress recognition in terms of accuracy, efficiency, and model interpretability.

## Introduction

### Implications of mental stress

We often encounter *stress* in daily life with variations of intensity and prolongation. Stress is understood as the response of the human body to mental and/or physical stimuli that involves the nervous system and hypothalamus-pituitary-adrenocortical axis^1^. According to the literature, stress is often classified as acute, episodic, or chronic^2^. Many contemporary studies have found stress to have a major impact on human health and cognitive performance. In some cases, stress has been shown to have a direct connection to depression, anxiety, stroke, cardiovascular disease, cancer, speech, and cognition impairment^3–5^. The negative effects of stress on human cognition are associated with dysfunctional changes in the prefrontal cortex and amygdala activation^5,6^, whereas the physical health effects of stress are related to detrimental changes in immunity and physical homeostasis^7^. As the complexities of human life increase, the effects of stress have begun to burden nations and, the globe at large^2,8^, which highlights the requirement for more research in this area. Early detection of harmful stress can be crucial as a part of effective stress management to promote greater wellbeing.

### Stress and electroencephalogram

Rapid development in sensor technologies and machine learning (ML) techniques have enabled research communities to begin to develop automated stress detection systems. These systems use invasive and/or non-invasive data acquisition methods. Stress recognition using invasive methods can be highly time-consuming and often require experts for data acquisition and processing^9–11^; this is not ideal for an automated system. The most common non-invasive methods include Electroencephalogram (EEG), heart rate variability, galvanic skin response, blood volume pulse, and electromyography for data acquisition^12^. Of these non-invasive methods, EEG is used most extensively for stress recognition due to its: information richness, cost-effectiveness, and high temporal resolution^13^.

### Stress recognition on the fly

Current methods for stress recognition use traditional ML techniques such as Linear Discriminant Analysis^14^, Naive-Bayes^15^, Support Vector Machine^16^, K-Nearest Neighbor^17^, and Multi-layer Perceptron^18^. However, these methods are not capable of evolving and adapting to new information after training, preventing them from being used in an online setup^19^. Online learning typically uses real-world data that changes with time, thus the model is adaptive and learns as new data is fed into it over time. In contrast, most stress detection approaches presented in the literature use static data to train and test the model. They also typically employ interventions, to manipulate the data used to train and test the models, such as feature engineering methods. It is difficult to compare the performance of known stress detection models because the feature engineering and extraction approaches differ from one study to another. This lack of standards also means that the generalizability of the methods presented is questionable^20^. Moreover, these traditional methods require a high volume of labelled data for model training. Today, the emergence of wearable technologies has revealed the potential for personalized health applications, designed to detect stress. Such applications must meet certain conditions to be practical. Use of online learning to allow the model to adapt to change, capability to operate under low power and the need for low-resource utilization are among them. This work focuses on finding solutions for the challenges posed by these conditions.

### Data drifts and online learning

One of the challenges in online learning is handling what is known as the drift phenomena successfully. Drifts can be observed in spatiotemporal data such as EEG and they can be defined in terms of input(s) and concept(s)^21^. The input(s) drift refers to the change of input data distribution over time without affecting the posterior probabilities of classes; concept drift refers to the change of posterior probabilities of the classes over time without any changes in the input distribution^22^. The drift phenomena require ML techniques to be able to acquire new knowledge without forgetting the prior knowledge (i.e., avoiding catastrophic forgetting) and even to update prior knowledge based on that new or recently gained knowledge. Adding to the challenge are the restrictions posed by online learning which demands the algorithm to use only a limited amount of pre-allocated memory, process a sample only once, use a consistent amount of time for processing, produce a valid model at each processing step, and perform in par with batch mode learning^19^.

### Spiking neural networks (SNNs)

SNNs are a class of artificial neural networks (ANNs) that are considered to be biologically plausible^23^. They have proven to be highly efficient in terms of time and memory requirements for data processing compared to commonly used sigmoidal counter parts^23^. The temporal dimension used in data processing is a major factor that contributes to their increased efficiency when compared with traditional ANNs, which makes SNNs an ideal candidate for online learning^24^. Moreover, the unsupervised learning mechanisms in SNNs have demonstrated capability in fast and data-efficient learning^25–27^. These attributes have led to the development of several online learning algorithms using SNNs with both supervised and unsupervised learning^21,28–37^. Of these methods, only a few algorithms use structural adaptation (i.e., evolving and pruning neurons and connections). Structural adaptation is crucial for learning new knowledge and forgetting irrelevant information in an online setup^21,29,34,35,37^. However, some of these structurally adaptive methods are built for batch mode learning only^29,37^ or do not fully exploit the temporal dynamics through learning^21,34,35^.

#### The online neuroplasticity spiking neural network (O-NSNN)

**Figure 1:**
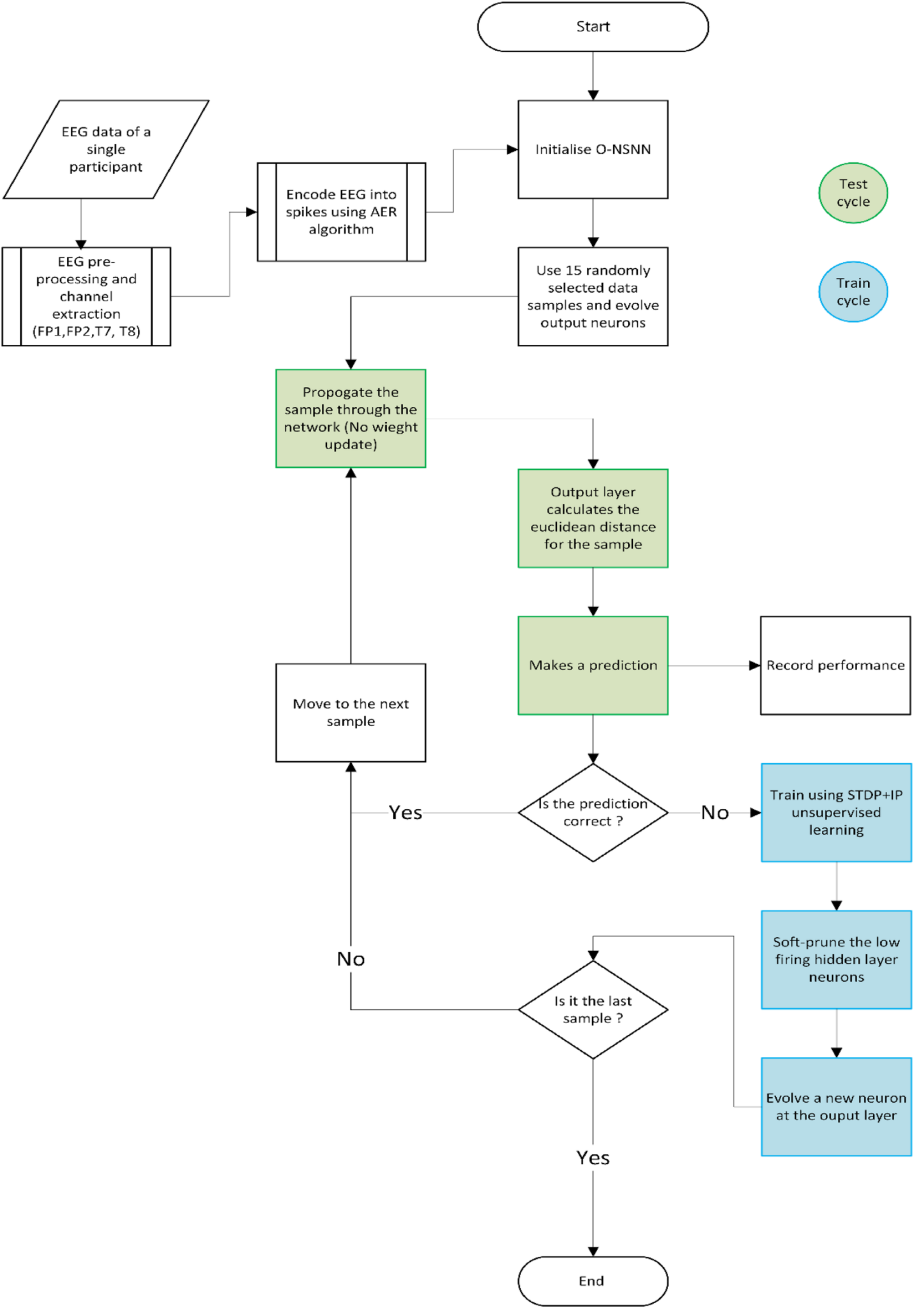
Flow diagram of the experiment. The experiment is conducted according to test-then-train regime^22^. Under this regime, the network is only trained when a prediction is incorrect.

The O-NSNN introduced in this work uses mathematical abstractions of selected plasticity techniques found in brain functions to fully exploit spatiotemporal patterns present in the data. This does not mean that the model mimics the entire neurobiological process of the brain, but rather it uses selected concepts of signal encoding, propagating, processing, and learning found in the brain. This algorithm differs from the previous ASNNs^21,29,34,35,37^ due to the inclusion of a full repertoire of plasticity techniques for temporal learning. These techniques are Spike Time Dependent Plasticity (STDP)^38^, Intrinsic Plasticity(IP)^39^, Neuron Evolving (neuron addition)^40^ and Neuron Pruning (neuron elimination)^41^. We hypothesize that this algorithm (see Fig. 1) will produce stable and faster pattern separation capability in the online classification of stress-related EEG by considering and handling the challenges associated with online learning.

**Figure 2:**
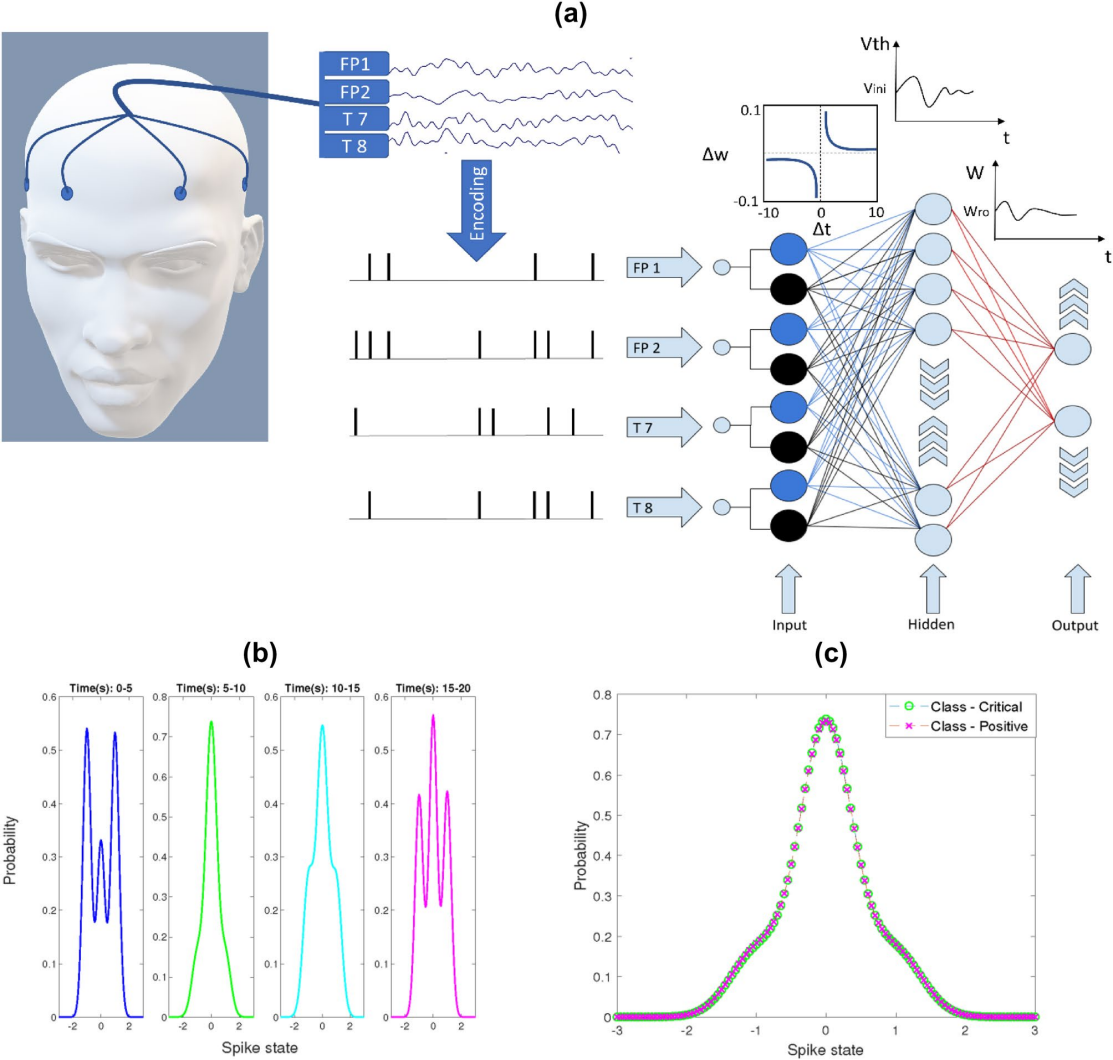
(**a**) The proposed O-NSNN architecture for stress recognition. EEG originating from FP1, FP2, T7 and T8 channels are encoded into spikes (using the AER algorithm) and propagated through a three-layered SNN architecture. An STDP rule is used for temporal learning between the input layer and the hidden layer. Hidden layer neurons use IP to adapt excitability based on the incoming data. The output layer learns using RO and SDSP rules. Each hidden layer neuron prunes itself according to soft-pruning rule and, the output layer evolves. (**b**) Stress class input samples of P1 with different spike rate distribution (Input drift) (**c**) Two separate classes of P1 (Critical and Positive) with the same input spiking distributions (Concept drift).

The proposed O-NSNN consists of three layers of Leaky-Integrate and Fire neurons (LIF)^42^ (see Fig. 2); a mathematical abstraction of a biological neuron that has demonstrated a greater balance between biological plausibility and computational tractability^43^. Before processing, the EEG signals are converted to their spiking equivalent using Address Event Representation (AER); a spike encoding algorithm used in artificial retina^44^. Thereafter, the first layer of neurons propagates spikes to the second layer via excitatory (blue) and inhibitory (black) synapses. During this propagation, the synaptic weights are updated using the STDP rule^38^. In addition, all the neurons adjust their excitability using an IP rule^45^. This combination of unsupervised STDP and IP prevents the network from getting caught up in a potentiation loop^46^, ensuring homeostasis^47^ and helping neurons extract independent spiking features from the input^48^. Moreover, the second layer of neurons undergoes a self-pruning process induced by error monitoring to avoid misclassifications caused by low-spiking neurons^45^. The synapses from the second layer to the third layer are excitatory and, follow a similar weight updating strategy discussed in dynamically evolving SNN (deSNN)^49^ that can evolve new neurons in the presence of new knowledge. However, unlike in deSNN, output neurons are not merged based on weight vector similarities (i.e., calculated using Euclidean distance of the input weight vector of a given neuron). In the presence of data drift, neurons of similar Euclidean distances may represent different classes. Therefore, we do not merge neurons rather, we eliminate or preserve neurons created based on the classification errors made during the data processing (Please refer to the Methods section for an in-depth explanation). This combined process of neuron addition in the third layer and, neuron pruning in the second layer are unique implementations that have not been discussed together in the published literature, to the best of our knowledge.

### Acute stress and data collection

The dataset used in this study consists of EEG recordings from 22 healthy participants (twelve males—average age = 27.92 years, standard deviation (σ) = 3.09 and ten females—the average age of 25.9 years, σ = 8.20) across three different conditions. On each condition, the participants were asked to listen to one type of comment, either critical, neutral, or positive. Such critical comments stimulate the part of the human auditory system of which the primary objective is to alert and warn^50^. Moreover, audio criticism has also been shown to induce mental stress levels in previous studies^51–53^ and music to induce positive and negative emotions^54^. Based on these previous studies, we presumed that the critical audio comments would induce acute stress in the participant. The details of these comments used for this study have been validated and published reviosuly^55,56^. In addition to EEG data, the perceived stress of each participant was recorded using the PSS-14 scale^57^. Each EEG recording lasted for two minutes, and the recordings were segmented into five-second splits to feed the O-NSNN. Consequently, a single sample of EEG data consisted of 1280 time points and four channels. From each participant, 72 such samples, with 24 samples for each class of stressed, neutral, and positive, were processed. Complete details of the dataset are given in the methods section.

#### EEG channels and performance measures

For the experiments of this study, we extracted signals from the FP1, FP2, T7, and T8 channels. In a previous study, researchers showed the sufficiency of two frontal channels for stress vs non-stress classification^58^. Furthermore, since the stimuli were auditory, T7 and T8 channels were used to capture the dynamics of the auditory cortex. Classification accuracy and sensitivity (true positive rate for stress EEG) was used to measure the performance. These measures using O-NSNN were compared against 70/30 split batch learning and online learning without structural plasticity (SP). For all experiments, we used individualized O-NSNN models since the effects of stress are found to be depending on an individual’s neurobiological predisposition^2^. Moreover, we used the prequential accuracy metric to evaluate the performance of online learning^59^. Secondly, these individualized models were subjected to an exploratory analysis that was undertaken to test the interpretability of the model and see if relationships could be discovered between acute and participant’s perceived stress.

This exploratory analysis involved comparing the personalized network activations to individually reported perceived mental stress levels. We categorized participants into one of three classes based on their PSS-14 scores (see Table 1). The connection weights of personalized models and Euclidean Distances (ED) of third-layer neurons were analyzed to find patterns within and between the perceived mental stress groups.

**Table 1:** Participant categorization according to perceived stress (PSS-14 score).

Label	PSS-14 score	Number of participants
High stress (HS)	PSS > 30	6
Medium stress (MS)	20 < PSS ≤ 29	11
Low stress (LS)	0 < PSS ≤ 19	5

In this work, we present a spatiotemporal data processing method for mental stress recognition and elucidate the possibility of investigating brain activity at an individual level. Therefore, the contribution of this study benefits both computer science and psychology/neuroscience research communities. The contributions of the study are as follows:

1. O-NSNN algorithm equipped with a biologically plausible repertoire of plasticity techniques for online mental stress recognition.
2. Insights into how perceived stress relates to incidences of acute stress.

## Results

We compared the classification accuracy and sensitivity of the O-NSNN model with the same learning framework without structural plasticity (SP) techniques (denoted as O-RSNN) and batch mode learning without SP (B-RSNN) (i.e., 70% of the samples for training and 30% for testing). The task involved measuring the accuracy of classifying EEG data into one of three possible classes: stress, neutral or positive conditions and the sensitivity (true positive rate) to recognize correctly classified stress instances. Since the synaptic weights of the first layer to the second are initiated randomly following Gaussian distribution, each experiment was conducted 30 times, allowing the accuracy and sensitivity to be reported statistically. The performance is presented in terms of average accuracy and sensitivity in Table 2. Furthermore, we explored patterns in network dynamics for knowledge extraction.

**Table 2:** Accuracy and sensitivity comparison between online [with SP (O-NSNN) and without SP (O-RSNN)] and batch mode (B-RSNN) learning.

Participant ID	O-NSNN		O-RSNN		B-RSNN	
	Accuracy	Sensitivity	Accuracy	Sensitivity	Accuracy	Sensitivity
P1	0.94 ± 0.02	0.93 ± 0.05	0.66 ± 0.09	0.50 ± 005	0.84 ± 0.07	0.76 ± 0.12
P2	0.93 ± 0.03	0.93 ± 0.20	0.57 ± 0.07	0.49 ± 0.09	0.72 ± 0.08	0.75 ± 0.10
P3	0.91 ± 0.06	0.96 ± 0.70	0.60 ± 0.06	0.59 ± 0.14	0.69 ± 0.09	0.61 ± 0.15
P4	0.90 ± 0.08	0.88 ± 0.04	0.77 ± 0.11	0.67 ± 0.22	0.93 ± 0.05	0.96 ± 0.07
P5	0.91 ± 0.06	0.80 ± 0.06	0.66 ± 0.11	0.61 ± 0.27	0.83 ± 0.09	0.81 ± 0.14
P6	0.92 ± 0.03	0.90 ± 0.45	0.79 ± 0.08	0.72 ± 0.18	0.88 ± 0.08	0.83 ± 0.12
P7	0.90 ± 0.06	0.63 ± 0.04	0.59 ± 0.08	0.62 ± 0.05	0.74 ± 0.09	0.82 ± 0.11
P8	0.94 ± 0.02	0.95 ± 0.09	0.68 ± 0.11	0.66 ± 0.25	0.88 ± 0.09	0.90 ± 0.09
P9	0.94 ± 0.02	0.94 ± 0.07	0.76 ± 0.08	0.79 ± 0.06	0.75 ± 0.10	0.96 ±0.70
P10	0.91 ± 0.06	0.95 ± 0.34	0.42 ± 0.07	0.48 ± 0.13	0.49 ± 0.11	0.53 ± 0.18
P11	0.91 ± 0.07	0.96 ± 0.17	0.75 ± 0.08	0.64 ± 0.11	0.86 ± 0.08	0.84 ± 0.14
P12	0.91 ± 0.04	0.93 ± 0.23	0.68 ± 0.08	0.76 ± 0.05	0.86 ± 0.08	0.81 ± 0.14
P13	0.92 ± 0.08	0.90 ± 0.06	0.82 ± 0.09	0.89 ± 0.21	0.92 ± 0.06	0.95 ± 0.07
P14	0.89 ± 0.07	0.86 ± 0.47	0.53 ± 0.10	0.54 ± 0.11	0.62 ± 0.10	0.60 ± 0.13
P15	0.92 ± 0.04	0.93 ± 0.09	0.66 ± 0.09	0.75 ± 0.14	0.77 ± 0.10	0.79 ± 0.16
P16	0.91 ± 0.04	0.93 ± 0.12	0.46 ± 0.10	0.36 ± 0.25	0.60 ± 0.12	0.71 ± 0.13
P17	0.86 ± 0.13	0.95 ± 0.05	0.49 ± 0.10	0.45 ± 0.21	0.71 ± 0.10	0.79 ± 0.16
P18	0.85 ± 0.09	0.87 ± 0.55	0.55 ± 0.09	0.55 ± 0.09	0.65 ± 0.09	0.62 ± 0.13
P19	0.90 ± 0.07	0.91 ± 0.06	0.41 ± 0.08	0.51 ± 0.15	0.61 ± 0.08	0.60 ± 0.16
P20	0.91 ± 0.07	0.95 ± 0.05	0.74 ± 0.10	0.64 ± 0.18	0.81 ± 0.08	0.87 ± 0.19
P21	0.90 ± 0.12	0.93 ± 0.04	0.64 ± 0.09	0.64 ± 0.05	0.75 ± 0.09	0.62 ± 0.11
P22	0.92 ± 0.03	0.87 ± 0.13	0.67 ± 0.09	0.53 ± 0.11	0.82 ± 0.09	0.89 ± 0.12

### Increased accuracy and robustness in O-NSNN

The highest average accuracy for O-NSNN was 93.63% for P1 and, the lowest was 85.29% for P18. The average accuracy across all participants was recorded at 90.91%, 63.18% and 76.04% for O-NSNN, O-RSNN and B-RSNN, respectively, whereas the average sensitivity was recorded at 90.27%, 60.86% and 77.36%. The O-NSNN outperformed O-RSNN across all 22 participants. In comparison, B-RSNN was outperformed in terms of accuracy by O-NSNN except for one participant (P4). Regarding sensitivity, the B-RSNN outperformed the O-NSNN with the data of P4, P5, P7, P9 and P22.

The performance of the O-NSNN was also compared with the most relevant studies that used a common data source, the DEAP dataset^60^, to classify stress vs relaxed brain signals (two classes). Here the O-NSNN recorded lower accuracy performance compared to batch mode experiments of SVM^61^ and SNN^29^ as shown in Table 3.

**Figure 3:**
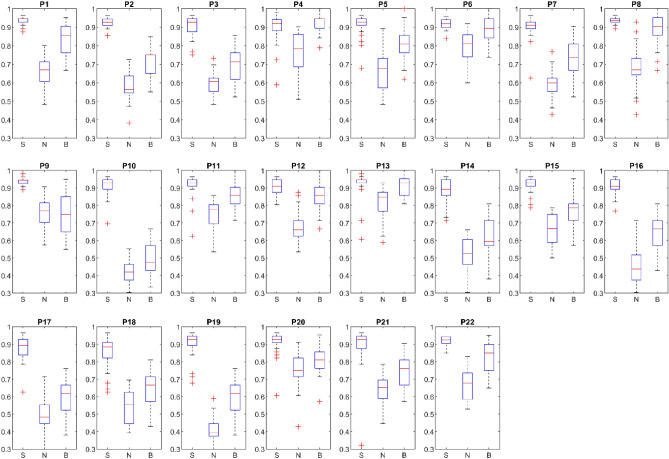
Performance variation of individual models. Performance distribution obtained from 30 testing cycles. At each cycle the initial weights between the input to hidden layers are selected pseudo randomly according to gaussian distribution. *S* Online learning with SP, *N* Online learning without SP, *B* Batch mode learning without SP.

Figure 3 shows the variation of performance for personalized models for each participant obtained from 30 pseudo-random network initiations. Accordingly, for all 22 participants, the O-NSNN model had the lowest degree of performance variation.

### The efficiency of O-NSNN

**Figure 4:**
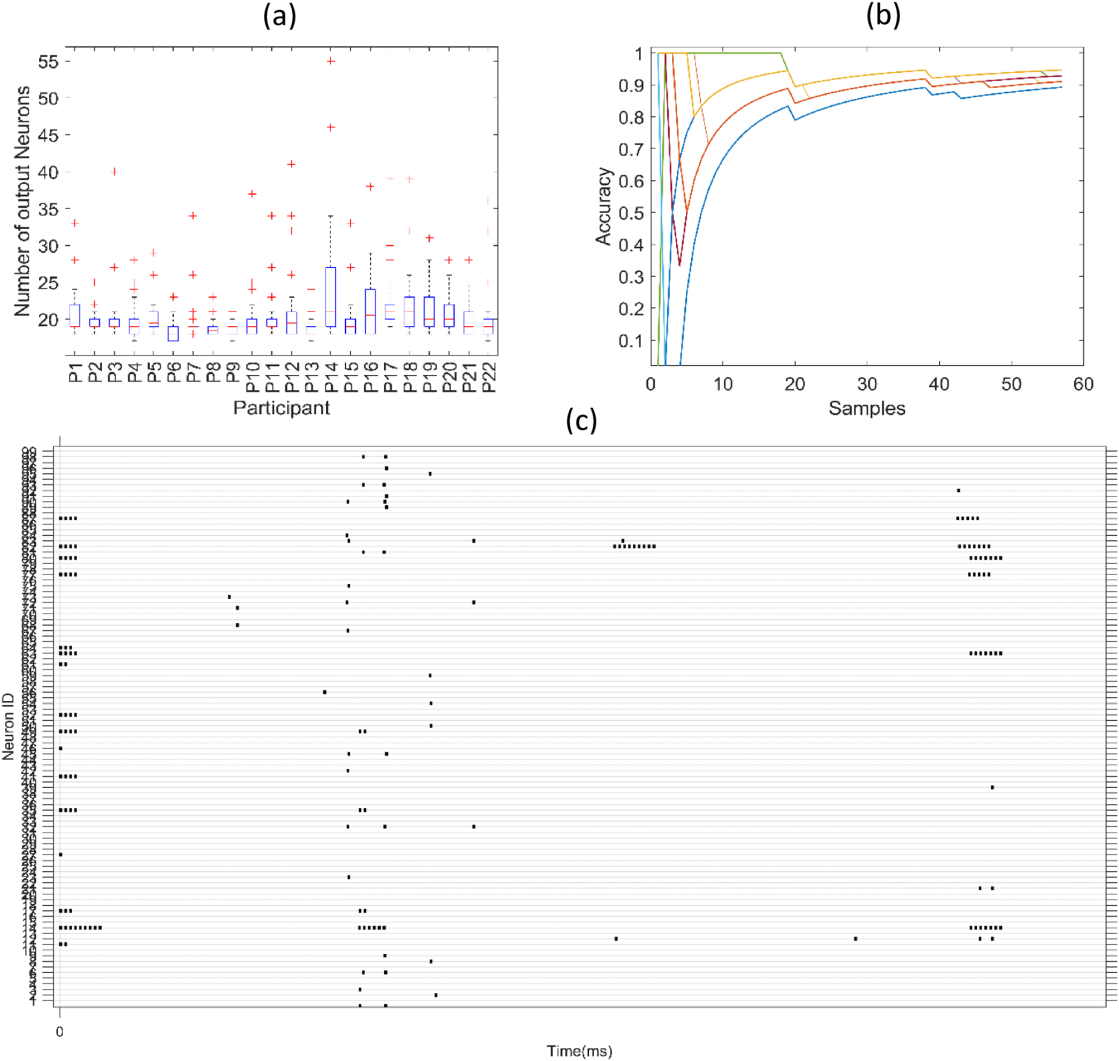
(**a**) Number of output neurons evolved by O-NSNN during 30 testing cycles for each participant model (**b**) Prequential accuracy progression with the number of samples increasing (**c**) Sample spiking raster plot of the hidden layer for P1.

The efficiency factor of the O-NSNN can be presented in terms of the number of output neurons used and spikes generated in the hidden layer. When the number of output neurons used was investigated, the O-NSNN method used, on average 20.39 (σ = 3.84) neurons (see Fig. 4a), whereas O-RSNN used 72 (i.e., absence of structural plasticity created a neuron for each input sample) and, B-RSNN used 50 output neurons respectively (i.e., 70/30 split training used 50 input samples for training where a neuron was created for each input). The spike generation of O-NSSN was measured as a ratio between the number of spikes received at the hidden layer to the number of spikes generated by the hidden layer, where the mean was recorded at 0.063 (σ = 0.009). This spike encoding is epitomized in Fig. 4c where the raster plot indicates the temporal sparseness of the spikes. When considering the trend of model accuracy over time, O-NSNN typically reached a prequential accuracy of 80% within 150 to 200 s of data processing commencement (the accuracy behavior against number of samples processed is given in Fig. 4b). An exception to this trend was noted in the case of P17 and P21 O-NSNN models.

### O-NSNN knowledge extraction

We also analyzed the Euclidean distance (ED) of the output neuron weight vectors and input to the hidden layer synaptic weights (i.e., STDP weights), of each individualized O-NSNN model. The evolved output neurons of an individualized O-NSNN model represented a certain class (i.e., stress, neutral or positive). The O-NSNN used this weight vector of the output neurons to predict the class of the incoming signals. Therefore, each ED of a sample is a numerical representation of the individual's brain signal under a given stimulus. Similarly, the weights of input to the hidden layer in O-NSNN are updated in an unsupervised method using STDP and IP. Once all the data samples are passed through the network, the O-NSNN weights (i.e., input to the hidden layer) capture the spatiotemporal correlations of the input signals.

#### Comparing numerical representations of brain signals

**Figure 5:**
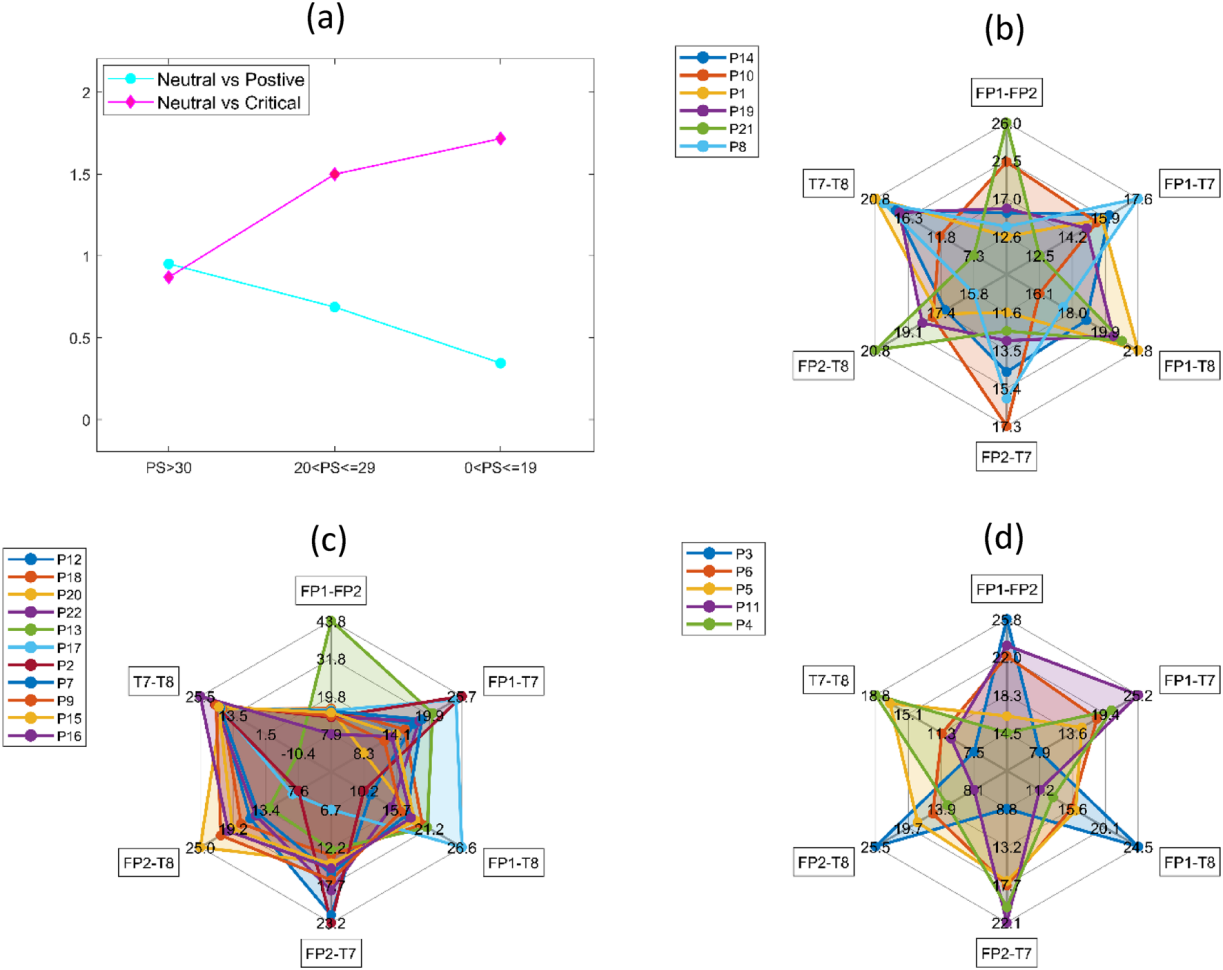
(**a**) Average differences between EEG samples represented by Euclidean distances. The signals during Neutral stimuli is selected as the baseline. (**b**) Spiking interaction pattern between channels for the High stress group (**c**) Spiking interaction pattern between channels for the Medium stress group (**d**) Spiking interaction pattern between channels for the Low stress group.

We compared the EDs between the HS, MS, and LS groups and found that the mean distance between neutral and critical stimuli of the HS group was 0.95 (σ = 0.41). In contrast, the LS group’s average distance between neutral and critical stimuli was much shorter at 0.25 (σ = 0.22). The average distance between neutral and positive stimuli of the HS group was 0.87 (σ = 0.86) and lower than that of the LS group’s distance of 1.86 (σ = 0.84). According to these results, the HS group’s EEG representations for positive stimuli did not differ to any notable extent from the EEG generated for neutral stimuli; this was the same for negative stimuli (i.e., under stress). However, the LS group recorded a much larger difference in both cases (see Fig. 5a).

#### Input channel correlation

**Figure 6:**
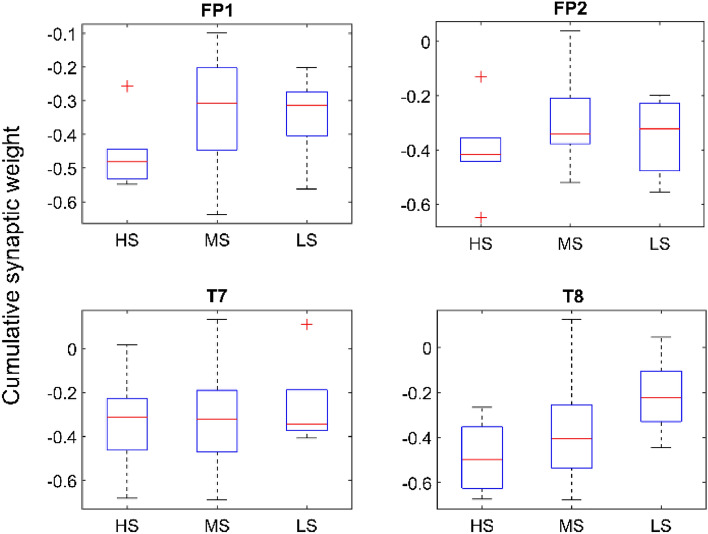
Cumulative weights of the synapses fanning out from respective inputs calculated according to perceived stress groups.

When considering the activations between input channels (i.e., using the input to hidden layer synaptic weights), the majority of MS participants exhibited similar activation patterns (see Fig. 5c), whereas the LS and HS groups exhibited irregular patterns of activation from one individual to another (see Fig. 5b,d). While investigating this further by examining the input synaptic weights of the hidden layer, we found that the HS group had higher inhibition than the LS group in the FP1 and FP2 channels (see Fig. 6). The same inhibitory patterns were observed for T8 but not T7. When examining the right and left-brain activations, we discovered that the HS group showed higher inhibition in the right hemisphere (FP2 and T8) than in the left hemisphere (FP1 and T7). However, in the LS group, the average difference between right and left hemisphere activations was significantly smaller. Moreover, higher activation was observed between FP1 and T8 than FP2 and T7 in five out of six participants in the HS group. The opposite activation pattern was observed in four out of five of the participants in the LS group.

## Discussion

This study presents Neuroplasticity Spiking Neural Network in an online learning setup for classifying the neural activity of healthy participants when exposed to comments that are intended to trigger different levels of mental states (i.e., stress, neutral, positive) and explores the link between these classifications and self-reported stress levels (perceived mental stress scores). This O-NSNN method produced higher pattern recognition capability on the fly, with increased efficiency, interpretability, and biological plausibility.

### The performance of the O-NSNN

**Figure 7:**
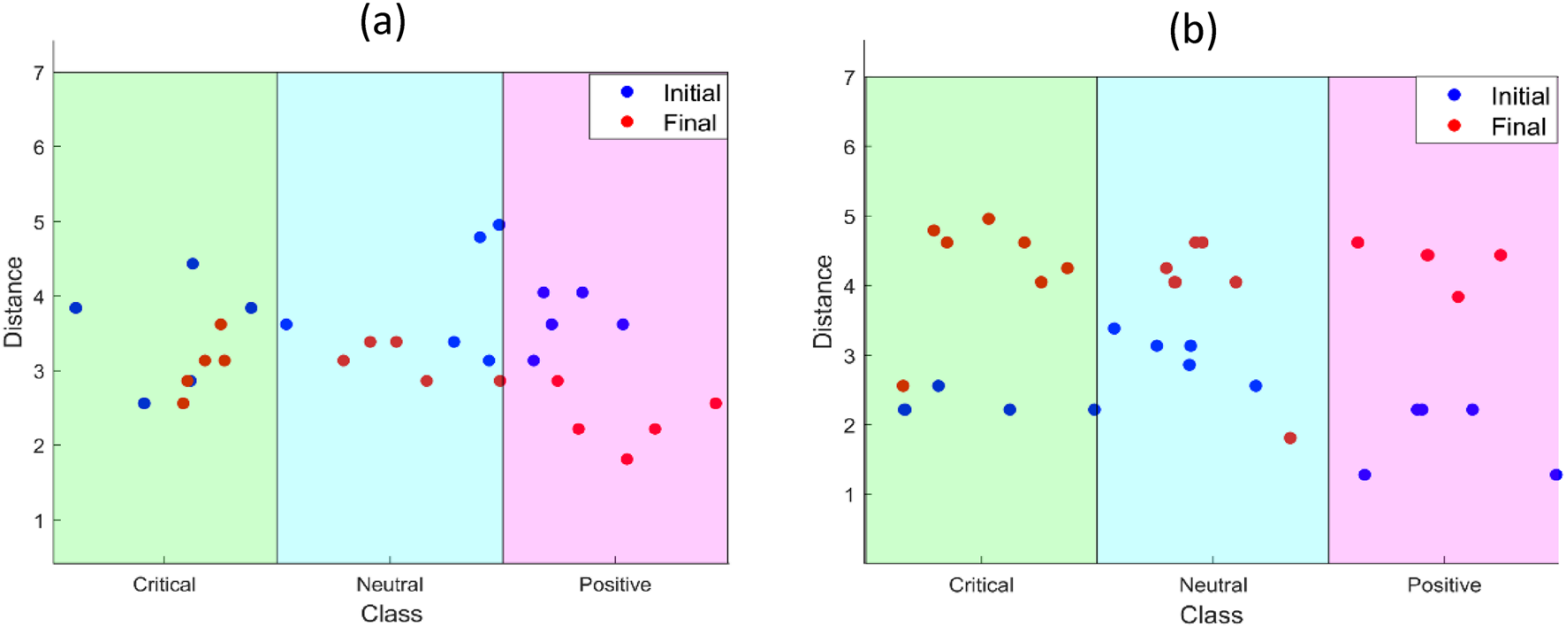
Euclidean Distance between initial(Blue) and final(Red) output neurons. The initiation process use the first 15 samples to evolve 15 output neurons. (**a**) without pruning or evolving new neurons (O-RSNN) (**b**) with pruning and evolving new neurons (O-NSNN).

The O-NSNN outperformed the other SNNs (O-RSNN and B-RSNN), in terms of average accuracy, as shown in Table 2. When comparing the two online learning methods, O-NSNN (90.76%, σ = 2.09) was found to perform significantly better than O-RSNN (63.08%, σ = 11.09) (*Student’s t-test, α* = 0.05,* p* = 0.005) in terms of accuracy. As per Fig. 3, the O-NSNN model produced the least performance variation indicating higher robustness^64^. When considering the DEAP dataset, the O-NSNN could not outperform SNN and SVM techniques built for stress recognition (Table 3). The methods that outperformed the O-NSNN used feature engineering^61^ or hyperparameter optimization^65^ methods for the modelling tasks. Exploring the modelling mechanisms of O-NSNN, we found that the EDs of output neurons (i.e., numerical representations of input samples) to have better discriminative capability between the initial and final states of O-NSNN than in O-RSNN. This enhanced discriminative capability is presented in Fig. 7 for P1. With neurons evolving and self-pruning being the only difference between O-NSNN and O-RSNN; we propose this SP technique as a successful method for handling new classes and/or new representations of already-known classes. In other words, the O-NSSN approach is effective at handling concept drift*.*

**Table 3:** Performance comparison with other studies that used the same data for stressed vs relaxed brain signal classification.

Study	Method	Accuracy	Sensitivity
Bastos-Fiho et al.^62^	K-NN (batch mode)	0.70	–
Shon et al.^63^	K-NN (batch mode)	0.72	
García-Martínez et al.^61^	SVM (batch mode)	0.81	–
Weerasinghe et al.^29^	SNN (batch mode)	0.92 ± 0.02	–
This study	NSNN (online mode)	0.80 ± 0.05	0.79 ± 0.04

#### STDP and IP learning

In a previous study, it was reported how hidden layer neuron pruning with STDP + IP leads to increased robustness and efficiency of SNNs in a batch learning setup for EEG classification^45^. In the same study, hidden layer neurons with low firing probability causing classification errors were noted. In this study, instead of completely pruning these low-firing probability neurons, we have adopted a self-pruning method that stops a neuron activation for a limited period. This is achieved by increasing the neuron threshold voltage to the highest value found in the population. The advantage of this method is three-fold. Firstly, the inactivity of the neuron caused by threshold alteration help in reducing the number of dimensions used to represent an input sample at the output layer. Since classifications of the proposed O-NSNN are based on EDs calculated from output layer synaptic weights, part of the increase in performance may be attributed to the mitigation of the *curse of dimentionality*^66^*.* Secondly, the self-pruned neurons remain in the network to respond to salient features that may occur due to drifts or new data. This repurposing of neurons may account for the improvement of the performance of the network with time^41^. Thirdly, the efficiency of this pruning is superior to regular synaptic pruning, which requires scanning of the entire weight matrix against a threshold^41,67^.

#### The efficiency of O-NSNN

The efficiency of the O-NSNN in terms of the number of neurons used and spikes generated reduced drastically with the use of STDP + IP learning and self-pruning. Unlike continuous streams of spiking, these techniques enabled sparser spiking activity resulting inactive states most of the time (see Fig. 4c). When compared to STDP-only learning, STDP + IP was shown to have reduced the average spiking by 35 times (*Student’s t-test, α* = 0.05,* p* = 0.008). This reduction of spikes minimizes the calculations involved from the hidden to the output layer. Moreover, the O-NSNN output layer utilized 3.52-times and 2.45-times lesser neurons on average compared to O-RSNN and B-RSNN models, respectively. In comparison to the early methods of evolving neurons where the spiking is not regulated^35,68^ and the output repository grows indefinitely^37^, this method is much more suitable for memory-restricted applications.

### Knowledge extraction

From trained O-NSNN models, HS participants showed lower activation levels in prefrontal channels FP1 and FP2 compared to the LS group. This was observed during the synaptic weight analysis of individual models, where the HS group had more inhibitory weights connected to FP1 and FP2 channels (see Fig. 6). Moreover, the T8-connected synapses showed higher activations for the HS group (compared to the LS group), but this was not the case with T7-connected synapses (see Fig. 6). In terms of the channel activation patterns, a similarity was observed among the individuals of the MS group but not in HS and LS groups (Fig. 5b–d). In addition, the HS group had the smallest difference between EDs (numerical representations of spike patterns) produced during stress and positive stimuli compared to neutral states, whereas in the LS group, the observation was the opposite (Fig. 5a). This suggests a lack of change in functional patterns of the brain to external stimuli in the HS group and, a greater change in functional patterns in the LS group. This observation leads to an interesting hypothesis about the relationship between acute and perceived stress. Namely, the individuals with high perceived stress (HS group) have less discrimination between positive and negative stimuli. In a previous study, long-term stress has been found to alter the perception of emotional stimuli^69^.

### Biological plausibility

The biological plausibility of O-NSNN can be discussed in the aspects of data processing techniques employed and the spiking behavior observed. Firstly, the data processing techniques inspired by neuroscientific concepts include STDP for temporal synaptic learning^38^, IP for neuron spike regulation^39^, self-pruning (apoptosis) to selectively restrict activation of neurons^70^, and addition of new neurons (neurogenesis) for retention of new knowledge^71^. Secondly, the model introduced demonstrates avalanche-like spiking which is also found in neocortical circuits^72^. Arguably this makes O-NSNN much more biologically plausible than other online learning methods introduced, which do not utilize the same repertoire of plasticity techniques or show spiking behavior close to what is found in biology^21,34,35^.

## Conclusion

This work presents a novel neural network algorithm for mental stress classification using EEG data and online learning. The algorithm adapts to individuals and uses functional concepts of the biological brain to learn, on the fly, in a resource-efficient manner. The O-NSNN algorithm introduced displayed superior performance in terms of accuracy, robustness, and resource efficiency over models that did not use structural plasticity.

Our method introduced goes beyond traditional black box ANN models to reveal insights into individual brain dynamics for better interpretation. Improving the capability of this algorithm to recognize a higher number of classes under resource restrictions could potentially contribute to the applications of wearable technology for the detection and monitoring of mental stress.

## Methods

### Neuroplasticity spiking neural network

Here we present a description of the O-NSSN model and the experimental framework designed to test the model. The NSNN is a fully connected, feed-forward spiking neural network consisting of LIF neurons^42^. The input nodes can process both excitatory and inhibitory spikes. These nodes are connected to the hidden layer via excitatory and inhibitory synapses in which the weights are updated using an unsupervised STDP learning algorithm^38^. The hidden layer neurons operate in an adaptive threshold scheme in an unsupervised manner using an IP learning rule^45^. The hidden layer is connected to the output layer via excitatory synapses updated according to Spike Driven Synaptic Plasticity^73^ and, initiated using the Rank Order (RO) rule^74^. The hidden layer neurons undergo a self-pruning mechanism. The third layer acts as the classifier and can evolve new neurons. All the hyperparameter values of the NSNN introduced are given in Table 4.

**Table 4:** O-NSNN hyperparameters.

Participant identifier		Online learning with SP
AER encoder	$\mathrm{f}$	0.7
LIF	${\mathrm{v}}_{\mathrm{thresh}}$	0.05
	${\mathrm{v}}_{\mathrm{rest}}$	0
	$\mathrm{R}$	1
	$\mathrm{C}$	10
STDP	${\mathrm{A}}_{+}$	0.001
	${\mathrm{A}}_{-}$	0.001
	${\uptau }_{\mathrm{pos}}$	10
	${\uptau }_{\mathrm{neg}}$	10
	${\mathrm{w}}_{\mathrm{max}}$	0.5
	${\mathrm{w}}_{\mathrm{min}}$	− 0.5
IP	${\uptheta }_{\mathrm{pos}}$	0.001
	${\uptheta }_{\mathrm{neg}}$	0.000001
Pruner	${\mathrm{sp}}_{\mathrm{thresh}}$	1
Classifier	$\mathrm{\alpha }$	1
	$\mathrm{mod}$	0.8
	$\mathrm{drift}$	0.001

#### Spike encoding using address event representation

AER is a biologically inspired spike encoding mechanism used in artificial retina applications^44^. Its simplicity, efficiency, and adaptiveness make it an attractive option for online applications. The temporal difference ${\mathrm{tempdiff}}_{(\mathrm{t})}$[refer Eq. (1)], between two temporarily contiguous data points (denoted ${\mathrm{x}}_{\mathrm{t}}$ and ${\mathrm{x}}_{\left(\mathrm{t}-1\right)}$) and, a user defined threshold factor $\mathrm{f}$ is used to calculate an adaptive spike threshold at each time step [refer to Eq. (2)]. If the EEG voltage value of the current time step is more than the threshold, an excitatory spike is emitted otherwise an inhibitory spike is emitted.

1 $${\mathrm{tempdiff}}_{(\mathrm{t})}={\mathrm{x}}_{\mathrm{t}}-{\mathrm{x}}_{\left(\mathrm{t}-1\right)}$$

2 $$\mathrm{threshold}=\mathrm{mean}\left({\mathrm{tempdiff}}_{\left(\mathrm{t}\right)}\right)+ \left(\mathrm{f}*\mathrm{std}\left({\mathrm{tempdiff}}_{\left(\mathrm{t}\right)}\right)\right)$$

#### Leaky integrate and fire neuron

The LIF neuron is commonly used in machine learning applications due to its computational tractability and the ability to produce basic spike behaviors^43^. Since this study involves an IP (adaptive voltage threshold) method, a wider variety of spiking behaviors can be produced than can be produced by a normal LIF^43^. The membrane potential change $\frac{{\mathrm{dv}}_{\mathrm{t}}}{\mathrm{dt}}$ of a LIF neuron can be modelled using a resistor–capacitor circuit and mathematically expressed using Eq. (3). In the equation, the time constant ${\uptau }_{\mathrm{m}}$ is equal to the product of resistance $\mathrm{R}$ and capacitance $\mathrm{C}$. The membrane potential is given by ${\mathrm{v}}_{\mathrm{t}}$ and, the input current at time $\mathrm{t}$ is given by ${\mathrm{I}}_{\mathrm{t}}$. The resting voltage of the neuron is given by ${\mathrm{v}}_{\mathrm{rest}}$.

3 $${\uptau }_{\mathrm{m}}\frac{{\mathrm{dv}}_{\mathrm{t}}}{\mathrm{dt}}={\mathrm{v}}_{\mathrm{rest}}- {\mathrm{v}}_{\mathrm{t}}+{\mathrm{RI}}_{\mathrm{t}} , {\uptau }_{\mathrm{m}}=\mathrm{RC }$$

#### Unsupervised learning

In the O-NSSN, the unsupervised weight update strategy STDP^38^ is accompanied by an IP rule^45^ that adapts the threshold of hidden layer neurons individually. This combination of plasticity is a key factor in maintaining firing homeostasis and enhancing SNN performance in terms of classification accuracy and efficiency^45,47,75^.

4 $$\mathrm{F}\left(\Delta \mathrm{t}\right)={\mathrm{A}}_{+} {\mathrm{exp}}^{(-\Delta \mathrm{t}/{\uptau }_{\mathrm{pos})}} \Delta \mathrm{t}>0$$

5 $$\mathrm{F}\left(\Delta \mathrm{t}\right)=-\mathrm{A}\_ {\mathrm{ exp}}^{(\Delta \mathrm{t}/{\uptau }_{\mathrm{neg})}} \Delta \mathrm{t}<0$$

6 $$\Delta {\mathrm{w}}_{\mathrm{ij}}=\sum_{\mathrm{a}}^{\mathrm{b}}\sum_{\mathrm{p}}^{\mathrm{q}}\mathrm{F}({\mathrm{t}}_{\mathrm{j}}^{\mathrm{m}}- {\mathrm{t}}_{\mathrm{i}}^{\mathrm{n}})$$

Equations (4) and (5) represent STDP according to Long-Term Potentiation (LTP) and Long-Term Depreciation (LTD) respectively^38^. Both equations are functions of the time difference $\Delta \mathrm{t}$ between spikes. In Eq. (6) the pre-synaptic neuron is denoted by $\mathrm{i}$ and the post-synaptic by $\mathrm{j}$. If $\mathrm{j}$ fires before $\mathrm{i}$, $\Delta \mathrm{t}$ is positive leading to LTP. A reversed firing sequence leads to LTD. In Eqs. (4) and (5), the positive and negative time constants are given by ${\uptau }_{\mathrm{pos}}$ and ${\uptau }_{\mathrm{neg}}$ respectively. These time constants are predetermined windows of time used for synaptic modifications. ${\mathrm{A}}_{+}$ and $\mathrm{A}\_$ terms determine the maximum synaptic modification. The cumulative weight change $\Delta {\mathrm{W}}_{\mathrm{ij}}$ is calculated using the spike timing of each pre-synaptic neuron from $\mathrm{p}$ to $\mathrm{q}$ and each post-synaptic neuron spiking from $\mathrm{a}$ to $\mathrm{b}$. The instantaneous spike time of each post-synaptic neuron is given by ${\mathrm{t}}_{\mathrm{j}}^{\mathrm{m}}$ and each pre-synaptic neuron by ${\mathrm{t}}_{\mathrm{i}}^{\mathrm{n}}$.

The IP rule operates simultaneously with STDP according to the two equations defined in (7). Here, the first expression of Eq. (7) is used to upregulate the neuron voltage thresholds and, the second to down-regulate.

7 $${\mathrm{v}}_{\mathrm{thr}}(\mathrm{t})=\left\{\begin{array}{c}{\mathrm{v}}_{\mathrm{thr}}\left(\mathrm{t}-1\right)+N{\uptheta }_{\mathrm{pos}}{\mathrm{v}}_{\mathrm{init}}, s\left(\mathrm{t}-1\right)=1\\ {\mathrm{v}}_{\mathrm{thr}}\left(\mathrm{t}-1\right)- N{\uptheta }_{\mathrm{neg}}{\mathrm{v}}_{\mathrm{init}}, otherwise\end{array}\right.$$

The threshold voltage of a neuron at time $\mathrm{t}$ is given by ${\mathrm{v}}_{\mathrm{thr}}(\mathrm{t})$. If the neuron fired in the previous time step and satisfies the condition $\mathrm{s}\left(\mathrm{t}-1\right)=1$, then a fraction of the initial voltage ${\mathrm{v}}_{\mathrm{init}}$ is added to the threshold voltage of the previous time step ${\mathrm{v}}_{\mathrm{thr}}\left(\mathrm{t}-1\right)$. This fraction is calculated using the product of the positive learning rate ${\uptheta }_{\mathrm{pos}}$ and the number of neurons in the hidden layer $\mathrm{N}$. If a spike did not occur in the previous time step, then the threshold voltage is lowered using the negative learning rate ${\uptheta }_{\mathrm{neg}}$. The two learning rates are determined based on the highest neuron activation and lowest information entropy^45^ after each sample propagation.

#### Structural plasticity

The addition of new neurons in the output layer and self-pruning of the hidden layer are the two key SP techniques incorporated in the NSNN algorithm. There are no neurons in the output layer at first. During the initiation process, a predefined number of neurons evolved. The number of samples used to evolve these initial neurons was 15 for the NSNN in this study. This set of neurons remains in the network and gets their weights updated at each sample pass. Since the NSNN operates under the test-then-train regime, if an error is made during the test phase, a new neuron is evolved in the following training phase. Here, an error symbolizes the emergence of a new class or a representational change in an already known class caused by concept drift^76^. Moreover, self-pruning also takes place in the hidden layer if an error is identified in the previous time step. This self-pruning is executed on neurons with low spiking probability since they can cause poor generalization^45^.

8 $${\mathrm{W}}_{\mathrm{jk}(\mathrm{init})}=\mathrm{\alpha }. {\mathrm{mod}}^{\mathrm{order}(\mathrm{j},\mathrm{k})}$$

9 $${\mathrm{W}}_{\mathrm{jk}}\left(\mathrm{t}\right)={\mathrm{W}}_{\mathrm{jk}\left(\mathrm{init}\right)}+\sum_{\mathrm{t}=1}^{\mathrm{n}}\mathrm{d}$$

The synaptic weights from the hidden to the output layer are initiated according to the RO rule given in Eq. (8). The initial weight between $\mathrm{j}$ pre-synaptic neuron and $\mathrm{k}$ post-synaptic neuron ${\mathrm{W}}_{\mathrm{jk}(\mathrm{init})}$, is determined using a learning parameter $\mathrm{\alpha }$ and an exponent of $\mathrm{mod}$. The modulation factor $\mathrm{mod}$ is determined based on the importance of the spike order. For the first spike to arrive at the synapse, $\mathrm{order}(\mathrm{j},\mathrm{k})$ starts at 0, thereby allocating the highest possible weight and increases as the spikes arrive at other neurons (i.e., decreases ${\mathrm{W}}_{\mathrm{jk}(\mathrm{init})}$). Thereafter, a drift parameter $\mathrm{d}$ is used to increase or decrease the initial weight to form a weight value at time $\mathrm{t}$, ${\mathrm{W}}_{\mathrm{jk}}\left(\mathrm{t}\right)$.

### Performance evaluation

To evaluate the performance in online learning, we used the prequential accuracy metric^76^ with the test-then-train approach^22^. In test-then-train, a sample is tested first before training. This method minimizes the memory cost since samples need not be held in memory. By applying prequential memory with this approach, accuracy can be updated incrementally. The accuracies for online learning stated in the study are the final accuracy performance after 360 s or 72 samples.

10 $${\mathrm{ACC}}_{\mathrm{pre}}\left(\mathrm{t}\right)=\left\{\begin{array}{c}{\mathrm{Acc}}_{\mathrm{pre}}\left(\mathrm{t}\right), if t={\mathrm{t}}_{\mathrm{init}}\\ {\mathrm{Acc}}_{\mathrm{pre}}\left(\mathrm{t}-1\right)+\frac{{\mathrm{Acc}}_{\mathrm{pre}}\left(\mathrm{t}\right)-{\mathrm{Acc}}_{\mathrm{pre}}(\mathrm{t}-1)}{\mathrm{t}-{\mathrm{t}}_{\mathrm{init}}+1}, else\end{array}\right.$$

In Eq. (10), the classification accuracy of the NSNN at time $\mathrm{t}$ is given by ${\mathrm{ACC}}_{\mathrm{pre}}\left(\mathrm{t}\right)$. Here, ${\mathrm{t}}_{\mathrm{init}}$ represents the initial time point which is taken as the reference time point. For the batch learning experiments (i.e., B-RSNN), we used the standard accuracy metric which is defined as the ratio of the number of correct predictions over the total number of predictions^77^.

### Ethics approval and consent to participate

All experiments were performed in accordance with the relevant guidelines and regulations. The Auckland University of Technology Ethics Committee (AUTEC) provided approval for the study on 2nd October 2019 (Approval identity number: 19/231). All participants were provided with a detailed informed consent form, which was also explained verbally, detailing the objectives, activities and consequences related to the study. All participants provided the signed informed consent form prior to data collection.

### EEG Data

The participant group consisted of 12 males with an average age of 27.92 (σ = 3.09) and 10 females with an average age of 25.9 (σ = 8.20). The EEG data were recorded over three sessions in a sound-attenuated room with a gap of at least one day between each session to prevent carry-over effects. At each session, the participant followed a sequence of steps: starting with completing the PSS-14 survey, recording two minutes of resting EEG, recording EEG while listening to an audio of either critical, neutral or positive comments, followed by a recording of two minutes of resting EEG. The type of audio comments for the session was selected randomly. Each comment lasted from 10 to 15 s and 40 such comments were made to listen through earphones during each session. It was presumed that critical comments would induce stress based on the result of previous studies^51–53^. However, it is noted that all participants may not be stressed to the same level by critical audio comments in an experimental setup. Therefore, the sensitivity to each comment was assessed using measurements of arousal and relevance on an 11-point Likert scale.

The 120 auditory comments used for the study were recordings of male and female native English speakers specifically trained to emphasize critical, neutral and positive comments through pitch and tone^55,56^. The critical and positive comments were typical remarks that one would hear from a close family member, and the neutral comments were factual statements that had no relevance to the participant. Samples of such comments include, “you are lazy and never finish anything you start! you’ve had chances but didn’t go through with it” (Critical comment); “you are good at organising things and paying attention to detail.” (Positive comment); “the Emu is the largest native bird in Australia, with long neck and legs” (Neutral comment). Details of these comments have been published previously^55,56^.

EEG recording was performed with a SynAmps amplifier and a 62-channel QuickCap with electrodes configured in the international 10–20 system. Electrodes channels were: FP1, FPZ, FP2, AF3, AF4, F7, F5, F3, F1, FZ, F2, F4, F6, F8, FT7, FC5, FC3, FC1, FCZ, FC2, FC4, FC6, FT8, T7, C5, C3, C1, CZ, C2, C4, C6, T8, TP7, CP5, CP3, CP1, CPZ, CP2, CP4, CP6, TP8, P7, P5, P3, P1, PZ, P2, P4, P6, P8, PO7, PO5, PO3, POZ, PO4, PO6, PO8, CB1, O1, OZ, O2, CB2. Data was recorded at 1000 Hz. Using multiple electrodes is a better approach than using a single electrode when assessing multiple levels of stress^58^. However, processing all the channels will require greater processing power. Therefore, FP1, FP2, T7 and T8 specific electrodes were selected. The selection of frontal electrodes were based on a previous EEG feature selection study conducted on stress classification which reported higher accuracy levels with FP1 and FP2^58^. Moreover, since the stress stimulations were auditory, T7 and T8 were used in an attempt to capture the dynamics of the auditory cortex. Previously, emotional auditory stimuli had been found to evoke different levels of valence in individuals that co-varied significantly with EEG signals generated by the auditory region^78^ and, negative valence is found to be strongly connected with stress^8^.

EEG data preprocessing was performed in MATLAB 2019a (The Mathworks, Inc)^79^ using custom scripts that involved functions from EEGLAB plugin^80^. Data were down-sampled offline to 256 Hz. A high-pass finite impulse response (FIR) filter at 0.01 Hz and a low-pass FIR filter at 50 Hz were applied. A baseline correction was not applied separately since the high-pass filter with low cutoff frequencies are found to rectify the baseline drift^81^. Using the CleanLine function^80^, line noise was removed before data were manually inspected for the removal of bad channels (flat or extremely noisy). The removed channels were interpolated before an independent component analysis was performed, to decompose the sample, using the *runica* function^80^ from the MATLAB ICA Toolbox for Psychophysiological Data Analysis^82^. The independent components derived from ICA were inspected and muscular and ocular artifacts were removed from the data based on their activity spectra and scalp topographies. After the preprocessing steps, the last five seconds of the voltage signal was selected (Each original EEG signal consisted of 10 to 15 s. i.e., the stimulus presentation time). This extracted portion of the voltage signal was then converted into temporal spikes using AER protocol^44^ before feeding the SNNs. No other feature engineering or extractions were carried out.

## Data Availability

The main dataset used in the current study is available from the corresponding author on reasonable request.
